# The Effect of Acclimation to Sublethal Temperature on Subsequent Susceptibility of *Sitophilus zeamais* Mostchulsky (Coleoptera: Curculionidae) to High Temperatures

**DOI:** 10.1371/journal.pone.0159400

**Published:** 2016-07-27

**Authors:** Jianhua Lü, Huina Zhang

**Affiliations:** Province Key Laboratory of Transformation and Utilization of Cereal Resource, Henan University of Technology, School of Food Science and Technology, Henan University of Technology, Lianhua Street, Zhengzhou High-Tech Development Zone, Zhengzhou, 450001, Henan, China; USDA-ARS, UNITED STATES

## Abstract

Heat treatment is a popular alternative to synthetic pesticides in disinfesting food-processing facilities and empty grain storages. *Sitophilus zeamais* Mostchulsky is one of the most cosmopolitan and destructive insects found in empty grain storage facilities and processing facilities. The effect of acclimation in *S*. *zeamais* adults to sublethal high temperature on their subsequent susceptibility to high temperatures was investigated. *S*. *zeamais* adults were acclimated to 36°C for 0 (as a control), 1, 3, and 5 h, and then were exposed at 43, 47, 51, and 55°C for different time intervals respectively. Acclimation to sublethal high temperature significantly reduced subsequent susceptibility of *S*. *zeamais* adults to lethal high temperatures of 43, 47, 51, and 55°C, although the mortality of *S*. *zeamais* adults significantly increased with increasing exposure time at lethal high temperatures. The mortality of *S*. *zeamais* adults with 1, 3, and 5 h of acclimation to 36°C was significantly lower than that of *S*. *zeamais* adults without acclimation when exposed to the same lethal high temperatures. The present results suggest that the whole facility should be heated to target lethal high temperature as soon as possible, avoiding decreasing the control effectiveness of heat treatment due to the acclimation in stored product insects to sublethal temperature.

## Introduction

The maize weevil, *Sitophilus zeamais* Mostchulsky (Coleoptera: Curculionidae), is a primary insect pest of cereal grains, particularly in maize and wheat, whose infestation usually starts in the field before harvest and extends in bulk grain and processing facilities [1–3]. Control of stored-grain insect pests has ever been primarily achieved by continued applications of methyl bromide and phosphine. However, methyl bromide has been phased out due to its ozone depleting potential according to the Montreal Protocol in the world [4,5]. Currently, control of *S*. *zeamais* population is primarily dependent upon intensive use of phosphine [6]. However, its repeated use for decades has disrupted biological control by natural enemies and led to serious concerns about insecticide resistance, environmental contamination, pesticide residue in food, and lethal effects on non-target organisms [7–11].

Recently, the integrated pest management (IPM) concept encourages the development of a sustainable nonchemical method to effectively manage *S*. *zeamais*. As an environment-friendly and convenient method, heat treatment has been widely evaluated and applied to controlling insect pests in empty grain storages and processing facilities [12–16]. Usually, the target facility is gradually heated from ambient temperature to 50–60°C during heat treatment [13,17–20], which naturally makes the stored product insects experience acclimation to sublethal high temperature. Many studies have shown that acclimation can significantly affect the thermal tolerance of insects [21–23].

In addition, most studies have focused on the effects of constant elevated high temperatures on mortality of stored grain insects [18,19]. In comparison, the effects of acclimation to sublethal temperatures on the mortality of stored-product insects are poorly understood, and such information is important for developing effective heat treatment protocols, and understanding responses to thermal stress and the adaptive evolution in response to climate warming [24,25]. In current study, the hypothesis is that acclimation to sublethal temperatures can enhance the heat tolerance of *S*. *zeamais* adults and decrease their mortality. And the objective of this study is to evaluate the effect of acclimation to sublethal temperature on subsequent susceptibility of *S*. *zeamais* adults to lethal high temperatures.

## Materials and Methods

### Insects

*S*. *zeamais* was cultured in a controlled temperature and humidity chamber (27±2°C, 75±5% relative humidity and 12:12 L:D) without exposure to any insecticide at the Institute of Stored Product Insects of Henan University of Technology, Zhengzhou, China. The food media used were washed, sterilized whole wheat grains at about 13.5% equilibrium moisture content. The cultivar of the wheat used as a food media was Zhoumai 22. Healthy and 1-2-week old adults were randomly chosen for bioassays.

### Experimental protocol of acclimation to sublethal temperature

*S*. *zeamais* adults were randomly selected and put into empty plastic vials (twenty adults each plastic vial with a few of small holes for heat quick distribution), and then exposed to 36°C [21] for 0 (as a control), 1, 3, and 5 h as different acclimation times, respectively. Subsequently, the *S*. *zeamais* adults were respectively exposed to high temperatures of 43, 47, 51, and 55°C for varying periods, i.e. (1) 43°C for 165, 205, 245, 285, 325, 365, 405, and 445 min, (2) 47°C for 10, 20, 30, 40, 50, 60, 70, and 80 min, (3) 51°C for 2.0, 2.5, 3.0, 3.5, 4.0, 4.5, 5.0, and 5.5 min, and (4) 55°C for 40, 50, 60, 70, 80, 90, 100, and 110 s. The relative humidity lied in the range of 50–60% at all tested exposure temperatures. Upon completion of exposure to 43, 47, 51, and 55°C, the plastic vial was immediately opened and the treated adults were gently brushed into a glass jar containing whole wheat. Their mortalities were determined after 2 days. The adults were considered dead if no movement was observed when prodded with a camel’s hair brush. Three replicates were conducted. The total sample sizes are the following: 4 acclimation times × 4 high exposure temperatures × 8 exposure times = 128 treatments, and the total number of *S*. *zeamais* adults tested in the experiments was 128 (treatments)× 20 (*S*. *zeamais* adults) × 3 replicates = 7680.

Insects are ectotherms, and ambient temperature therefore significantly affects their life activities. The optimal temperatures are 25–33°C for growth and reproduction of most stored product insects. Our preliminary experiment results showed that the *S*. *zeamai*s adults could withstand a long-term heat stress before they eventually died at 36°C. Ma and Ma [21] acclimated two aphid species, *Sitobion avenae* and *Rhopalosiphum padi* at 36°C for testing their heat-escape temperatures. Meanwhile, heat treatment usually raises the ambient temperature of the target facility to 50–60°C, and the high exposure temperatures of 43, 47, 51, and 55°C frequently occur in the heating process. Thus, we selected 36°C as a short-term sublethal acclimation temperature, and 43, 47, 51, and 55°C as high exposure temperatures.

### Statistical analysis

*S*. *zeamais* adult mortality data after exposure to different high temperatures for varying periods were calculated as percentages. Mean ± SE mortality of the control adults in each combination of exposure temperature and exposure time was 0.0 ± 0.0. Therefore, treatment mortality data were not corrected for control mortality [26]. Treatment percentage mortalities were transformed to arcsine square-root values for two-way analysis of variance (ANOVA) procedure with insect mortality as response variable and acclimation time, and exposure time as fixed effects, and the mean mortalities were compared and separated by Scheffe’s test at *p* = 0.05. These analyses were performed using SPSS Version 16.0 software.

## Results

### The mortality of *S*. *zeamais* at 43°C

The mortality of *S*. *zeamais* adults significantly increased with increasing exposure time at 43°C (Table 1). Compared with the mortality of *S*. *zeamais* adults without acclimation (set as a control, 0 h), the mortality of *S*. *zeamais* adults with acclimation was significantly lower, especially when the exposure times were more than 365 min at 43°C. The acclimation time, exposure time, and the interaction between the acclimation time and exposure time significantly affected the mortality of *S*. *zeamais* adults at p<0.05 level (Table 2).

**Table 1:** The effect of acclimation to sublethal high temperature on mortality (%) of *S*. *zeamais* exposed to 43°C.

Exposure time /min	Acclimation time /h			
	0	1	3	5
165	3.3±1.67dA	5.0±2.89cA	0.0±0.00dA	1.7±1.67dA
205	5.0±2.89dA	3.3±3.33cA	3.3±1.67cdA	1.7±1.67dA
245	18.3±3.30dA	10.0±5.77cAB	6.7±3.33cdAB	1.7±1.67dB
285	41.6±11.67cA	15.0±7.64cB	13.3±3.33bcdB	8.3±4.41cdB
325	45.0±15.28cA	25.0±5.00bcAB	21.7±0.00bAB	10.0±6.67bcB
365	65.0±2.89bcA	38.3±8.82abB	30.0±8.66abB	20.0±2.89bB
405	86.7±7.26abA	40.0±8.66abB	26.7±4.41abB	21.7±4.41abB
445	95.0±5.00aA	53.3±9.28aB	35.0±1.67aB	33.0±5.00aB

Note: Data are Mean ± SE of three replicates. Different lowercases indicate significant differences in the same column, and different capital letters indicate significant differences in the same row (p<0. 05. The same as Tables 3, 5 and 7.

**Table 2:** Two way analysis of variance (ANOVA) parameters for main effects and associated interactions for the mortality of *S*. *zeamais* exposed to 43°C.

Source	df	Type III SS	Mean square	F-value	p-value
Acclimation time	3	15409.115	5136.372	81.841	0.000
Exposure time	7	31635.156	4519.308	72.009	0.000
Acclimation time × Exposure time	21	8101.302	385.776	6.147	0.000
Error	64	4016.667	62.760		
Total	96	117425.000			

### The mortality of *S*. *zeamais* at 47°C

The mortality of *S*. *zeamais* adults exposed to 47°C is listed in Table 3. The mortality of *S*. *zeamais* adults also significantly increased with increasing exposure time at 47°C. The mortality of *S*. *zeamais* with acclimation was significantly lower than that of *S*. *zeamais* without acclimation (control) to sublethal high temperature, especially when the exposure times were more than 40 min at 47°C. The acclimation time, exposure time, and the interaction between the acclimation time and exposure time significantly affected the mortality of *S*. *zeamais* adults at p<0.05 level (Table 4).

**Table 3:** The effect of acclimation to sublethal high temperature on mortality (%) of *S*. *zeamais* exposed to 47°C.

Exposure time /min	Acclimation time /h			
	0	1	3	5
10	1.7±2.89dA	0.0±0.00bA	0.0±0.00dA	1.7±1.67cA
20	3.3±2.89dA	0.0±0.00bA	0.0±0.00dA	1.7±1.67cA
30	16.7±12.58cA	5±2.89bA	7.7±2.41cdA	3.3±1.67cA
40	35.0±13.23bA	13.3±6.01bB	8.3±3.33cdB	6.7±3.33bcB
50	86.7±5.77aA	35.0±10.00aB	16.7±4.41bcdB	16.7±4.41bB
60	91.7±2.89aA	46.7±6.01aB	28.3±10.93bcB	24.3±5.33aB
70	91.7±5.77aA	36.7±7.26aB	36.7±9.28bB	31.7±4.41aB
80	93.3±2.89aA	55±13.23aB	42.2±6.41aB	37.6±6.82aB

**Table 4:** Two way analysis of variance (ANOVA) parameters for main effects and associated interactions for the mortality of *S*. *zeamais* exposed to 47°C.

Source	df	Type III SS	Mean square	F-value	p-value
Acclimation time	3	21202.865	7067.622	101.071	0.000
Exposure time	7	45397.906	6485.415	92.745	0.000
Acclimation time × Exposure time	21	12149.552	578.550	8.274	0.000
Error	64	4475.333	69.927		
Total	96	155003.000			

### The mortality of *S*. *zeamais* at 51°C

Table 5 shows the mortality of *S*. *zeamais* adults exposed to 51°C. The mortality of *S*. *zeamais* also significantly increased with increasing exposure time at 51°C. The mortality of *S*. *zeamais* with acclimation was significantly lower than that of *S*. *zeamais* without acclimation (control) to sublethal high temperature, especially when the exposure times were 2, 5, and 5.5 min at 51°C. The acclimation time and exposure time significantly affected the mortality of *S*. *zeamais* adults at p<0.05 level, and the interaction between the acclimation time and exposure time had no significant effect on the mortality of *S*. *zeamais* adults (Table 6).

**Table 5:** The effect of acclimation to sublethal high temperature on mortality (%) of *S*. *zeamais* exposed to 51°C.

Exposure time /min	Acclimation time /h			
	0	1	3	5
2.0	3.3±2.89cA	0.0±0.00bB	0.0±0.00bB	0.0±0.00bB
2.5	16.7±20.21bcA	1.7±1.67bA	8.3±14.43bA	6.7±1.67abA
3.0	25.0±25.98bcA	1.7±1.67bA	7.7±2.89bA	10.0±2.89abA
3.5	41.3±14.43abA	36.7±14.24abA	30.0±19.05abA	30.0±16.07abA
4.0	51.7±11.55abA	40.0±20.21abA	33.3±17.86abA	23.3±8.33abA
4.5	56.7±8.66bA	41.7±19.22abA	40.0±22.72abA	31.7±1.67abA
5.0	86.7±11.55aA	51.7±10.14abAB	52.3±12.55aAB	38.3±19.22aB
5.5	95.0±5.77aA	65.0±20.82aAB	60.0±16.00aAB	41.7±17.40aB

**Table 6:** Two way analysis of variance (ANOVA) parameters for main effects and associated interactions for the mortality of *S*. *zeamais* exposed to 51°C.

Source	df	Type III SS	Mean square	F-value	p-value
Acclimation time	3	7711.458	2570.486	8.219	0.000
Exposure time	7	45732.292	6533.185	20.889	0.000
Acclimation time × Exposure time	21	3838.542	182.788	0.584	0.915
Error	64	20016.667	312.760		
Total	96	178050.000			

### The mortality of *S*. *zeamais* at 55°C

Table 7 shows the mortality of *S*. *zeamais* adults exposed to 55°C. The mortality of *S*. *zeamais* also significantly increased with increasing exposure time at 55°C. The mortality of *S*. *zeamais* with acclimation was significantly lower than that of *S*. *zeamais* without acclimation (control) to sublethal high temperature, especially when the exposure times were 50, 60, 70, 80, 90, and 110 s at 55°C. The acclimation time and exposure time significantly affected the mortality of *S*. *zeamais* adults at p<0.05 level, and the interaction between the acclimation time and exposure time had no significant effect on the mortality of *S*. *zeamais* adults (Table 8).

**Table 7:** The effect of acclimation to sublethal high temperature on mortality (%) of *S*. *zeamais* exposed to 55°C.

Exposure time /s	Acclimation time /h			
	0	1	3	5
40	1.7±2.89dA	0.0±0.00bA	0.0±0.00cA	1.7±1.67dA
50	5.0±0.00dA	0.0±0.00bB	0.0±0.00cB	1.7±1.67dB
60	21.7±29.30cdA	20.0±17.56bA	3.3±3.33cB	1.7±1.67dB
70	31.7±24.66cdA	35.0±12.58bA	25.0±22.55bcAB	3.3±3.33dB
80	56.7±20.21bcA	33.3±10.92bA	28.0±9.23bcA	8.3±4.41dB
90	78.3±33.29abA	70.0±10.00aAB	65.0±7.64abBC	55.0±13.23cC
100	86.7±23.09abA	83.3±16.67aA	76.7±16.67aA	75.0±2.89bA
110	96.7±5.77aA	96.3±1.67aA	81.7±1.67aB	81.7±3.33aB

**Table 8:** Two way analysis of variance (ANOVA) parameters for main effects and associated interactions for the mortality of *S*. *zeamais* exposed to 55°C.

Source	df	Type III SS	Mean square	F-value	p-value
Acclimation time	3	4869.531	1623.177	6.153	0.001
Exposure time	7	106805.990	15257.999	57.839	0.000
Acclimation time × Exposure time	21	3382.552	161.074	0.611	0.896
Error	64	16883.333	263.802		
Total	96	272625.000			

## Discussion

The current study indicated that acclimation to sublethal high temperature could significantly enhance the survival of *S*. *zeamais* adults subsequently exposed to lethal high temperatures and reduce their mortality. In other words, acclimation to sublethal high temperature significantly enhanced the heat tolerance level of *S*. *zeamais* adults and reduced their subsequent susceptibility to lethal high temperatures.

The susceptibility of *S*. *zeamais* to lethal high temperatures was affected by various treatment factors, including insect strain, developmental stage, temperature-time combination, acclimation to sublethal high temperature, heating rate and treatment condition [27]. Li et al. [27] reported that the slowest heating rate (0.1°C/min) achieved the highest mortality of *S*. *zeamais* in controlled atmosphere conditions but lowest mortality in regular air conditions. In the present study, we investigated the effect of acclimation to sublethal temperature on subsequent susceptibility of *S*. *zeamais* adults to lethal high temperatures. The effect of acclimation on different strains and developmental stages (egg, larva, and pupa stages) of *S*. *zeamais* needs to be further investigated in the future.

The ability of insects to deal with heat stress can be achieved through physiological and biochemical mechanisms [28–31], including short-term processes such as acclimation or long-term processes such as evolutionary adaptation [32](Huey, 2010). Short-term heat acclimation in laboratory shapes part of the insect responses to their ambient environment, which may involve physiological and biochemical changes to cope with environmental temperature variation [29,33,34]. Tungjitwitayakul et al. [35] reported that heat shock at 30–50°C for 1 h increased three heat shock protein (hsp) genes expression in *S*. *zeamais* as follows: *Szhsp70* > *Szhsp90* > *Szhsc70*. In the present study, *S*. *zeamais* adults were acclimated at 36°C for 0 (control), 1, 3, and 5 h. Probably, the three *hsps* and other *hsps*, as well as some other metabolic regulation pathways, were responsible for the enhanced heat tolerance of *S*. *zeamais* adults with acclimation. Furthermore, when *S*. *zeamais* adults were exposed to 43, 47, 51, or 55°C for various time intervals, no significant differences were observed among the mortalities of *S*. *zeamais* adults with 1, 3, and 5 h of acclimation to 36°C, indicating that similar physiological and biochemical mechanisms were involved in the increased thermal tolerance of *S*. *zeamais* adults with acclimation. Therefore, the physiological and biochemical adaptation mechanisms of *S*. *zeamais* adults with acclimation necessarily deserves to be further investigated, which are in favor of understanding responses to thermal stress and the adaptation evolution in response to ongoing climate warming [25].

Generally, the temperatures are not evenly distributed in the whole treated facility during heat treatment. This inevitably results in the acclimation in stored product insects to sublethal temperature during heat treatment process, which will enhance their survival in the heat stress environment. The survived individuals will form a threat to the stored products after the heat treatment. According to the present research results, the whole target facility should be elevated to over 50°C as soon as possible to avoid reducing the disinfestation effectiveness resulting from the acclimation of stored product insects to sublethal temperature.
